# Parents’ Perspectives on Using Computerized Cognitive Training to Manage Cognitive Late Effects in Children With Cancer: Qualitative Descriptive Study

**DOI:** 10.2196/88656

**Published:** 2026-10-13

**Authors:** Tao Liu, Lin Mo, Feisal Hasen, Shishuang Zhou, Cho Lee Wong, XueMei Tang, Ting Han

**Affiliations:** 1 Department of Outpatient Children's Hospital of Chongqing Medical University Chongqing China; 2 School of Nursing Chongqing Medical University Chongqing China; 3 The Nethersole School of Nursing, Faculty of Medicine The Chinese University of Hong Kong Hong kong China; 4 Design Academy Sichuan Fine Arts Institute Chongqing China; 5 School of Design Shanghai Jiao Tong University Shanghai China

**Keywords:** parents, pediatric cancer, computerized cognitive training, CogniFit, cognitive late effects, qualitative study

## Abstract

**Background:**

Cognitive late effects affect 35%-60% of patients with pediatric cancer. Computerized cognitive training (CCT) is promising for cognitive training. Parents play a key role in children’s CCT participation, particularly by providing guidance and practical support during training. Yet, little is known about parents’ views on integrating CCT into the management of cognitive late effects in these patients.

**Objective:**

This study aimed to explore parents’ perspectives on the use of CCT for managing cognitive late effects in children with cancer, including perceived facilitators, barriers, and suggestions for improvement.

**Methods:**

We conducted semistructured interviews with parents of children with cancer aged 3-6 years who had used or were currently using CCT for cognitive training. Interviews were audio-recorded and transcribed verbatim in Chinese and analyzed using inductive thematic analysis. Selected anonymized quotations were translated into English by the first author and reviewed against the original Chinese transcripts to ensure accuracy and preserve contextual meaning.

**Results:**

We recruited 20 parents and identified 3 main themes and 12 subthemes regarding the use of CCT for cognitive management in children with cancer: (1) Perceptions of Integrating CCT into Cognitive Management, including Narrowing the Gap with Peers, Brief Yet Persistent Participation, It Freed Up My Time, Perceived Changes in Cognitive Functioning, and It Brings Fun; (2) Challenges, including Sometimes It’s a Bit Hard for the Child, Sometimes We Forget to Do the Training, Fear of the “Retard” Label, and Can We Afford to Keep Doing This?; and (3) Opportunities for Improvement, including Need for Tailored Training, Information Needs, and Don’t Leave Us Hanging.

**Conclusions:**

This study suggests that parents generally perceived CCT as a practical, engaging, and convenient approach to supporting cognitive management in children with cancer, particularly for home-based training. However, several challenges remain to be addressed to facilitate sustained engagement with CCT. Although some parents perceived improvements in their children’s cognitive functioning following CCT, these perceptions do not establish the effectiveness of the intervention. Overall, this study highlights the potential value of CCT from parents’ perspectives and provides practical insights for developing more flexible, individualized, and family-centered CCT interventions. Future research should incorporate children’s perspectives, recruit more diverse samples, consider potential concerns related to prolonged screen use, and use objective cognitive outcomes to evaluate CCT effectiveness.

## Introduction

Cancer and its treatment can result in cognitive late effects in children, some of which may persist into survivorship and may emerge or persist into adulthood [1,2]. Studies have shown that the incidence of such difficulties can be as high as 35%-60%, with potential impacts on activities of daily living [1]. Among survivors of childhood cancer, commonly reported long-term cognitive difficulties include declines in memory, attention, language expression, executive function, and processing speed [2-4]. These impairments may affect educational attainment, occupational achievement, and employment opportunities [5-7]. Survivors may therefore require long-term rehabilitation [1,8], and cognitive late effects can have substantial consequences for their long-term quality of life [9].

Several approaches have been used to manage neurocognitive difficulties associated with childhood cancer and its treatment, including pharmacological treatment, exercise training, and psychotherapy [10]. Although these approaches may improve aspects of cognitive functioning, their effectiveness and sustained participation may be influenced by various factors, including children’s age and developmental characteristics, individual needs, treatment-related factors, and potential adverse effects of pharmacological treatment [11-13]. These considerations highlight the need for cognitive interventions that are accessible, engaging, and adaptable to the individual needs of children with cancer.

Recently, computerized cognitive training (CCT) has received increasing attention as a potential intervention to support cognitive functioning in children with cancer [14]. Compared with traditional face-to-face cognitive training, CCT can provide diverse and structured cognitive training tasks and incorporate adaptive and gamified designs to adjust task difficulty according to children’s individual performance and provide immediate feedback, thereby potentially enhancing interactivity, enjoyment, and engagement [15,16]. In addition, CCT can be delivered remotely, providing more convenient opportunities for cognitive training for children living in areas with limited rehabilitation resources or those located far from medical centers [17,18]. These features may be particularly relevant for children with cancer, whose treatment schedules, clinical conditions, and treatment-related symptoms may limit their ability to participate regularly in hospital-based rehabilitation [18].

Previous studies of CCT and related computerized cognitive interventions among children and adolescents with cancer have predominantly used quantitative methods to evaluate cognitive outcomes, feasibility, and adherence [15,19,20]. However, quantitative measures may not fully capture children’s and families’ experiences of CCT or the facilitators, barriers, and support needs that influence its acceptability, sustained engagement, and fit within everyday family contexts [21,22]. Therefore, qualitative research from a stakeholder perspective can provide complementary insights into the practical experiences and contextual factors involved in CCT implementation and identify facilitators, barriers, and support needs that may not be adequately captured by quantitative measures [21,23].

Parents, as primary caregivers and decision-makers for children with cancer, also provide companionship, assistance, and support during home-based training [24,25]. Their perspectives are therefore important for understanding the acceptability and practical implementation of CCT. Previous qualitative research in pediatric oncology has similarly shown that parents’ perspectives can inform the design and implementation of digital health and other supportive interventions, including the identification of needs related to engagement, accessibility, individualized care, and ongoing support [26,27]. However, qualitative evidence regarding parents’ experiences, needs, and perceived barriers to implementing CCT for children with cancer remains limited.

Therefore, this study aims to explore the perspectives of parents of children with cancer on the integration of CCT into their children’s cognitive management, identify factors that facilitate or hinder the training process, and understand their feelings, needs, and preferences. The findings may inform the design and implementation of future CCT interventions that are responsive to the needs of children with cancer and their families.

## Methods

### Design Strategy

We conducted a descriptive qualitative study using semistructured interviews to explore parents’ perspectives and experiences regarding CCT for their children with cancer. The RATS (Relevance, Appropriateness, Transparency, and Soundness) framework was used to guide the reporting of this qualitative study [28]. RATS was selected because its core principles are well aligned with the descriptive and exploratory aims of this study, particularly its emphasis on understanding parents’ perspectives and experiences of their involvement in their children’s CCT program.

### Research Team

Our research team included both junior and senior investigators from medicine, computer science, and design disciplines. Several team members (TL, XT, and SZ) are clinicians with extensive experience working closely with patients with pediatric cancer and their caregivers over time. The team comprised both male and female researchers, bringing diverse professional perspectives to this study.

### Ethical Considerations

This study received ethical approval from the Ethics Committee of the Children’s Hospital of Chongqing Medical University (2023.288-1). Written informed consent was obtained from the children’s parents before they participated in this study. To protect participants’ privacy, each participant was identified by a unique code rather than their real name during data analysis. No financial compensation was provided for participation. All raw interview data and participant information were anonymized and stored on an encrypted cloud drive to ensure confidentiality.

### Participants

Parents of children with cancer aged 3-6 years were recruited using purposive and convenience sampling, consistent with the use of multiple sampling approaches in qualitative research [29]. Participants were recruited from 2 sources at a pediatric specialty hospital in Southwest China: the intervention group of a previously conducted randomized controlled trial (RCT; ChiCTR2400092143) investigating the effectiveness and feasibility of CCT in children with cancer, and the hospital’s pediatric oncology outpatient clinic.

For parents recruited from the RCT intervention group, children met the RCT eligibility criteria, including being aged between 3 and 6 years, having a cancer diagnosis confirmed by clinical pathology reports, having a full-scale IQ score of ≥70 on the Wechsler Preschool and Primary Scale of Intelligence–Fourth Edition to ensure sufficient cognitive capacity to understand and participate in the training program [30], and having a parent or legal guardian without severe illness or cognitive impairment who could understand this study’s procedures and support the child’s participation in the intervention and assessments. Parents of children who had received the CCT intervention were invited to participate in the qualitative interviews, and convenience sampling was used based on parents’ availability and willingness to participate. For outpatient recruitment, purposive sampling was used to recruit parents of children with cancer aged 3-6 years who had experience participating in or guiding their children in the CCT program and could communicate fluently in Chinese. Recruitment posters were displayed in the outpatient clinic, and interested parents completed a brief screening questionnaire before being contacted to arrange an interview.

Throughout the recruitment process, variation in parental educational attainment, children’s cancer type, treatment duration, and CCT experience was considered to capture diverse perspectives. Recruitment and interviews were conducted on a rolling basis. Of the 31 parents who initially agreed to participate, 6 were excluded for lacking prior CCT experience, 3 were unable to participate because of scheduling conflicts, and 2 withdrew without providing a reason. Ultimately, 20 parents participated, including 16 from the RCT intervention group and 4 from the outpatient clinic. Data saturation was reached by the 20th interview, with no new themes emerging thereafter, and recruitment was discontinued.

### Description of the CCT intervention

In the intervention group of the RCT, participants were instructed to complete cognitive training games for 30-45 minutes per day, 5 days a week, for 12 weeks. The intervention used the CogniFit cognitive training system [31], an off-the-shelf, standalone cognitive training software. CogniFit was selected because it provides game-based cognitive training targeting multiple cognitive domains and can be accessed through commonly available digital devices [32]. Before the intervention, the CogniFit software was installed on smartphones or tablets available to the participating families, allowing children to complete the CCT tasks at home. Parents were required to accompany their children throughout the intervention to facilitate task completion and provide necessary assistance. CogniFit supports 19 languages, including English, Spanish, Chinese, etc, enabling it to meet the needs of users from diverse linguistic backgrounds. The software has gained widespread recognition in the field of cognitive assessment and training, with its training content spanning multiple cognitive skill areas, including memory, attention, reasoning, and visuospatial ability (Figure 1).

At the beginning of the intervention period, participants received a 30-40 minute tutorial. Throughout the 12-week intervention, participants had access to support from therapists at any time. During this period, the first author (TL) conducted weekly telephone guidance sessions to monitor children’s participation and identify any difficulties, discomfort, or concerns related to the training.

**Figure 1:**
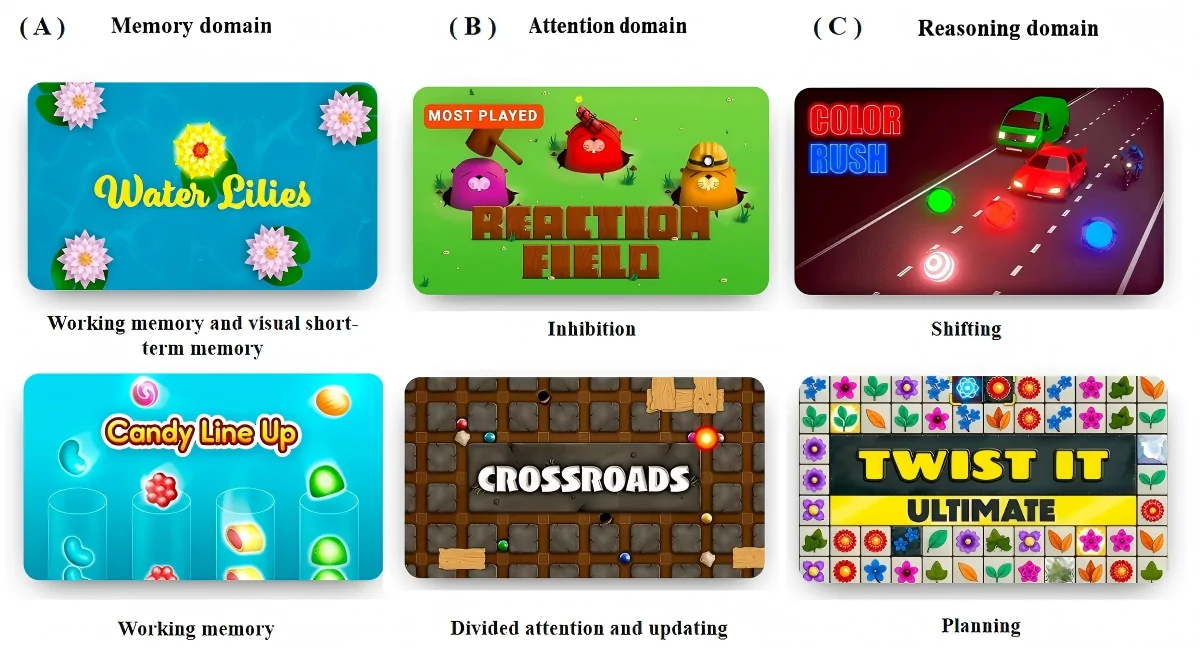
Part of the cognitive training games in Cognifit. A. Memory domain; B. Attention domain; C. Reasoning domain.

### (A) Memory Block

#### Water Lilies (Working Memory and Visual Short-Term Memory)

Remember the sequence in which flowers will be highlighted. During your turn, click on the flowers according to the order previously presented.

#### Candy Line Up (Working Memory)

The goal of the game is to fill the jars with the same type of candy. You may only place candies of the same type on top of each other.

### (B) Attention Block

#### Reaction Field (Inhibition)

Hit the moles of the target color as fast as you can. Avoid the moles with dynamites and double tap those with helmets.

#### Crossroads (Divided Attention and Updating)

Avoid balls colliding. If two balls come close to each other, quickly place a stone at the intersection. Use only the stones you need.

### (C) Reasoning Block

#### Color Rush (Shifting)

Your goal is to drive as fast as you can while collecting orbs of the same color as the vehicle.

#### Twist It Ultimate (Planning)

You must align tiles of the same type by exchanging adjacent tiles.

### Data Collection

Participants were recruited from the RCT intervention group and the outpatient clinic, and a total of 20 individual qualitative interviews were conducted following recruitment. For parents recruited from the RCT intervention group, interviews were conducted approximately 1 week after their children completed the 12-week CCT intervention, whereas interviews with parents recruited from the outpatient clinic were scheduled according to their availability. All interviews were conducted from June to July 2024, either in person or by telephone, according to participants’ preferences. In-person interviews were conducted in a meeting room at a specialized children’s hospital in Chongqing, the largest pediatric cancer center in western China, while telephone interviews were conducted remotely. The interviews were conducted by a doctoral student in nursing (TL), who had received training and had experience in conducting qualitative interviews. TL had no preexisting personal or clinical relationship with the participants before their enrollment in this study. Each interview lasted 30-40 minutes and was audio-recorded. Given TL’s dual role as both the intervention facilitator and qualitative interviewer, a reflexive audit was conducted throughout data collection and analysis. TL maintained reflexive notes to document potential researcher bias and its influence on participants’ responses. An audit trail was maintained to enhance the transparency and credibility of the research process. Although qualitative research does not prescribe fixed sample size requirements, widely accepted methodological guidance recommends determining sample adequacy based on data saturation [33]. Data collection and analysis were conducted iteratively, and data saturation was assessed based on whether subsequent interviews generated new themes or relevant information.

To develop the interview question guide, we first conducted a comprehensive review of relevant literature. Subsequently, we invited 2 parents of children with cancer to participate in a preliminary interview. After the research team’s review and adjustments, the final interview question guide was determined (Textbox 1). The interview guide questions were open-ended, covering participants’ feelings about the intervention, their experiences with using the CCT equipment, and the barriers and facilitators to participation. During the introduction of the interviews, we emphasized that there were no wrong answers and encouraged participants to express both positive and negative views regarding the use of CCT.

**Interview guide.**

Interview outline

- When you first heard about CCT, what were your initial thoughts and feelings?
- How do you perceive the effectiveness of CCT compared to traditional treatments?
- How was your experience using the CCT device? Do you think the CCT has been helpful for you and your child?
- During the process of using CCT, what challenges did you and your child encounter?
- What factors prompted you to continue using CCT?
- What suggestions do you have for the current CCT project?
- How can CCT be made more accessible and user-friendly for children with cancer?

### Data Analysis

All interviews were audio-recorded and then transcribed verbatim in Chinese. The transcribed data were deidentified, and each parent was assigned a unique participant identifier. Data were analyzed using an inductive thematic analysis approach guided by the 6-phase framework by Braun and Clarke [34]. The analysis involved (1) familiarization with the transcripts, (2) generation of initial codes, (3) searching for themes, (4) reviewing themes, (5) defining and naming themes, and (6) producing the report.

The first author (TL) and second author (FH) independently coded the transcripts and identified salient features of the data. The researchers then compared their coding structures and discussed similarities and differences in coding and interpretation. Disagreements were resolved through discussion and joint reflection until consensus was reached. Related codes were subsequently grouped into subthemes, which were reviewed and organized into broader main themes. The themes and subthemes were iteratively refined against the original transcripts to ensure that they accurately represented participants’ experiences. Representative anonymized quotations were selected to illustrate each theme and subtheme and retain participants’ perspectives. The final themes and subthemes were subsequently emailed to participants for member checking. Three participants provided feedback, primarily concerning the wording and organization of the subthemes. Their feedback was considered and incorporated into the final thematic structure where appropriate.

Importantly, all thematic analysis was conducted using the original Chinese transcripts to preserve conceptual accuracy and contextual meaning. Selected quotations presented in this paper were translated from Chinese into English by the first author (TL), a bilingual doctoral researcher proficient in English, and independently reviewed by the second author (FH) for linguistic accuracy and consistency with the original Chinese transcripts. Any discrepancies in the translated quotations were discussed and resolved by consensus, with particular attention to preserving the original meaning and contextual nuances of participants’ statements.

## Results

### Participant Characteristics

A total of 20 parents were recruited to participate in this study. Currently, all of these parents serve in the role of primary caregivers. A demographic overview of the children with cancer and their parents is provided in detail in Table 1.

**Table 1:** Demographic characteristics of participating parents and children with cancer.

Demographic characteristics				Values
**Parent**				
	Age (years), mean (SD)		32.96 (4.53)	
	**Sex** **,** **n (%)**			
		Female	14 (70)	
		Male	6 (30)	
	**Educational level,** **n (%)**			
		Primary	2 (10)	
		Secondary	10 (50)	
		Tertiary	8 (40)	
**Child**				
	**Sex,** **n (%)**			
		Female	8 (40)	
		Male	12 (60)	
	Age (years), mean (SD)		4.81 (1.03)	
	**Type of cancer,** **n (%)**			
		Leukemia	9 (45)	
		Brain tumor	5 (25)	
		Other	6 (30)	
	Duration of cancer treatment (years), mean (SD)		1.87 (0.69)	
	**Previous CCT^a^** **use, n (%)**			
		Yes	20 (100)	
		No	0 (0)	
	**Number of CCT programs used, n (%)**			
		1	14 (70)	
		2	3 (15)	
		≥3	3 (15)	
	**Frequency of CCT use, n (%)**			
		<3 times/week	7 (35)	
		3-5 times/week	12 (60)	
		>5 times/week	1 (5)	

^a^CCT: computerized cognitive training.

### Reflexive Insights

Reflexive reflection informed how the research team interpreted parents’ accounts of CCT, taking individual and family circumstances into consideration. For example, when parents described improvements in their children’s attention or task performance, the research team interpreted these accounts as subjective perceptions rather than evidence of intervention effectiveness. Similarly, missed or interrupted training sessions were understood in the context of competing caregiving demands, such as treatment schedules and hospital visits, rather than attributed solely to a lack of motivation. The research team also recognized that favorable accounts of convenience and engagement could coexist with difficulties involving task complexity and the need for parental assistance. These reflections supported an interpretation of parents’ experiences that acknowledged both perceived benefits and practical challenges within individual and family circumstances.

### Findings

Three main themes were identified to describe parents’ perspectives on the use of CCT for managing cognitive late effects in children with cancer. Each theme comprised several corresponding subthemes, as detailed in Textbox 2.

**Themes and subthemes of this study.**

**Perceptions on the integration of computerized cognitive training into cognitive management**

- 1a: Narrowing the Gap With Peers
- 1b: Brief Yet Persistent Participation
- 1c: It Freed Up My Time
- 1d: Perceived Changes in Cognitive Functioning
- 1e: It Brings Fun

**Challenges**

- 2a: Sometimes It’s a Bit Hard for the Child
- 2b: Sometimes We Forget to Do the Training
- 2c: Fear of the “Retard” Label
- 2d: Can We Afford to Keep Doing This?

**Opportunities for improvement**

- 3a: Need for Tailored Training
- 3b: We Wanted to Know More
- 3c: Don’t Leave Us Hanging

### Theme 1: Perceptions on the Integration of CCT Into Cognitive Management

#### Overview

This section presents our findings regarding the ongoing use of CCT among children with cancer, based on parents’ perspectives after implementation. The CCT based on CogniFit is deemed acceptable and feasible for both children with cancer and their parents. As 1 parent remarked, whereas previously purchased educational toys would be left untouched by their child, this app engaged him for 20 minutes at a time, with the ability to pause, making it well-suited to his short attention span.

#### 1a: Narrowing the Gap With Peers

Fourteen of the 20 parents expressed the perception that their children’s cognitive abilities, particularly attention and language, were weaker than those of their peers. These parents were concerned that such cognitive difficulties might persist or affect their children’s future development and were therefore willing to consider CCT as a potential training option.

I feel that his attention span isn't very good. He has difficulty concentrating to complete a task, and the teachers at school have also mentioned similar issues to me. That's why we want to take measures to change this situation.

Parent 11

When I compare her with other children of the same age, I feel that her language development is somewhat behind. I hope the training can help her improve so that she can communicate more like other children.

Parent 3

#### 1b: Brief Yet Persistent Participation

Multiple participants emphasized that conducting cognitive training on mobile phones is highly convenient, and one can start it anytime and anywhere if desired. As 1 parent put it, previously purchased educational toys were often left unused by children. In contrast, this application can continuously engage a child’s attention for 20 minutes and allows for pauses, which precisely aligns with the characteristics of children’s short attention spans.

Mobile-based training is highly convenient, unlike traditional cognitive training, which demands considerably more time...and we are willing to continue using this approach.

Parent 7

Half-hour training sessions are quite appropriate. Since the child regularly uses mobile devices anyway, it is preferable for him to engage with educational games like Cognifit that can capture his interest.

Parent 15

#### 1c: It Freed Up My Time

Participants described using CCT for cognitive training as highly convenient. The cognitive minigames in CogniFit featured adaptive difficulty levels, adjusting according to the child’s performance. This allowed parents to choose appropriate times and locations for training, which they perceived as more time-efficient than their previous experiences with face-to-face rehabilitation.

I find the training very convenient... The child can carry out the training by themselves, which eases our caregiving burden. I never thought playing games could have such benefits one day.

Parent 2

This modality is convenient and beneficial for many individuals, particularly those residing in remote areas, as it eliminates extensive travel time and saves considerable time.

Parent 13

This approach is still very time-saving. Unlike before, when I had to go to the hospital for rehabilitation every week, each visit would take up most of half a day.

Parent 18

#### 1d: Perceived Changes in Cognitive Functioning

Nearly all participants described changes they perceived in their children’s cognitive functioning during or following their use of CogniFit.

He (child) used to have great difficulty concentrating on a single task, often losing interest after just a few minutes. Now, it feels like his attention span has improved significantly.

Parent 17

Some participants perceived positive changes in their children’s cognitive functioning; however, they found it difficult to identify or describe specific improvements.

My husband says there's a difference. He's noticed some improvements... The child used to struggle a bit in this regard. But now he can complete a task immediately, though I can't recall exactly what it was.

Parent 19

#### 1e: It Brings Fun

All parents expressed that using gamified methods for cognitive rehabilitation training was highly feasible and enjoyable. Children generally demonstrated a strong interest and fondness for this game-based training approach. They further indicated that they also trusted this professionally screened game-based training method.

The child is quite interested in these mini-games... There are different difficulty levels and various games, and sometimes he can play on his own for a long time.

Parent 1

Compared to him playing those other games by himself, I much prefer him engaging in these educational mini-games... He's also quite interested in these.

Parent 8

### Theme 2: Challenges

#### 2a: Sometimes It’s a Bit Hard for the Child

Although all participants considered CogniFit easy to use and rarely encountered challenges, some participants reported encountering certain issues during the intervention. When the training tasks exceeded the children’s current abilities, they observed that the children would lose interest.

For my child, some of the games might be too difficult. For instance, the Mandala (a game in Cognifit)—I don't think it's suitable for such young children. When they couldn't understand it, they just threw the tablet aside.

Parent 6

When we started a new game, it would get a bit frustrating because the child didn't grasp the game rules, even though we had watched the instructional video.

Parent 17

#### 2b: Sometimes We Forget to Do the Training

In the family setting, persistence poses a major challenge for parents. Forgetting to conduct the training is not merely a simple oversight; rather, it reflects the conflict that parents face due to their multiple family roles (caregiver, worker, and household manager). Some participants indicated that in addition to managing their children’s cancer treatment, they also had to handle numerous household chores and caregiving responsibilities. This sometimes resulted in having no time or forgetting to use CCT.

People of our age have to take care of both the elderly and the young. Sometimes we're really so busy that we forget to have our child do the training.

Parent 7

I also have another child to take care of, and both of them are naughty boys. I simply can't spare any time for this training.

Parent 19

#### 2c: Fear of the “Retard” Label

During the participation process, 3 participants expressed concerns that their children might be labeled as “retarded” because of their use of CCT. They perceived cognitive training as something not typically associated with young children and worried that others might interpret their children’s participation in CCT as a sign of intellectual impairment.

If we use this for home-based training and the neighbors find out... They'll think the child has cerebral palsy. Before a week is up, the whole village will know.

Parent 3

Cause, in my understanding, this kind of thing (CCT) is for older adults with dementia... Our child is still so young. Do they really need this kind of training?... If others find out that our child has an intellectual problem, it would be very embarrassing.

Parent 11

#### 2d: Can We Afford to Keep Doing This?

Five participants reported financial difficulties. They mentioned that if it were not for the funding from this project covering the training costs, they would be unable and unwilling to pay for it.

Using this CCT costs several hundred yuan every month, and it can't be reimbursed through medical insurance. I don't think it's worth it because the child still needs treatment for their cancer.

Parent 2

We know this kind of software is quite expensive. There's no way around it. The child has to take medicine and see doctors every month. We'll have to wait a few years and see.

Parent 19

### Theme 3: Opportunities for Improvement

#### 3a: Need for Tailored Training

Participants expressed a need for CCT to be tailored to their children’s individual cognitive needs, interests, and daily circumstances. Some parents described selecting training tasks based on their children’s specific difficulties, while others suggested incorporating children’s interests and daily routines into the training design.

My child has difficulty maintaining attention. When I specifically selected more mini-games targeting attention for him, I felt that he made more progress. So, I think we could select different training programs according to each child's individual needs.

Parent 6

Could the training be integrated into the child's daily life and interests? For example, my child is a boy and might prefer racing games. It would be very helpful to have a training plan and related training scenarios tailored to his interests.

Parent 15

#### 3b: We Wanted to Know More

Participants expressed a need for more detailed and reliable information about CCT both before and during the training process. They felt that a better understanding of the training could help them feel more confident about participating in and continuing the training.

Actually, we don't know much about this training method at first. The researchers just gave us some explanations... but I only know it's beneficial for the child, and it would be great if there were more detailed explanations.

Parent 4

I also searched online for this type of game-based cognitive training. It seemed useful, but I wasn't sure whether the information I found was accurate. I wanted to know more about the training so that I could better understand whether it was suitable for my child.

Parent 16

#### 3c: Don’t Leave Us Hanging

For children with cancer in the rehabilitation phase, the intervals between check-ups can sometimes be quite long. During these periods, patients may encounter numerous issues while undergoing training and require detailed guidance from health care professionals. Excessive waiting times to have their problems resolved can undermine the effectiveness of the training. According to the participants, having the opportunity to consult with their doctors or researchers about current problems would be highly beneficial. This way, they can continue to follow up and adjust the treatment plan accordingly.

If there's one thing I deeply desire, it's to engage in frequent dialogues with professional experts.

Parent 2

Sometimes we feel really at a loss. We're not professionals and don't understand the underlying principles of this. It would be great if we could stay in touch with you.

Parent 20

## Discussion

### Principal Findings

This study explored parents’ perspectives on the use of CCT for cognitive management in children with cancer. Overall, parents viewed CCT positively and recognized its potential value as a convenient and engaging approach to cognitive management, yet these perceived benefits coexisted with multiple barriers to sustained engagement. These barriers reflected challenges at the levels of the training itself, children’s clinical and developmental characteristics, family circumstances, and broader social and economic contexts. Parents’ experiences also highlighted the need for CCT to be more responsive to individual cognitive characteristics and to provide greater informational and professional support for families.

### Parents’ Perceived Benefits and Digital Literacy in CCT Engagement

In this study, parents perceived CCT as entertaining and motivating and viewed it as a promising approach for supporting their children’s cognitive management. Similar positive perceptions of CCT have been reported among parents of survivors of childhood cancer, who generally rated the intervention as acceptable and were willing to recommend it to other families [29]. The perceived advantages of CCT were twofold. First, parents described the interactive features of CCT, including immediate feedback and dynamic difficulty adjustment, as potentially supporting children’s motivation and continued engagement [19]. Second, parents’ perceptions that their children experienced greater cognitive difficulties than their peers appeared to motivate their interest in seeking and trying cognitive interventions [24]. Compared with traditional face-to-face rehabilitation, some parents perceived CCT as more convenient and less time-consuming, particularly because it allowed their children to complete training at home and reduced the need for frequent hospital visits [18,29]. Previous studies have suggested that parents’ digital literacy and ability to support technology-based interventions may influence sustained use of CCT [25,35]. In light of our findings, future implementation of CCT may benefit from considering caregivers’ digital literacy, understanding of the intervention, and perceived barriers to use [35]. For caregivers with limited digital literacy but high acceptance of CCT, brief, practical, and scenario-based guidance may facilitate initial use and continued engagement. For caregivers with higher digital literacy but lower acceptance, clear evidence-based information and individualized feedback may help address concerns and strengthen confidence in the intervention [36]. These strategies may improve the feasibility and acceptability of CCT, although their effectiveness should be evaluated in future intervention studies. Future studies should use well-designed randomized controlled trials to determine whether the perceived benefits of CCT translate into measurable improvements in cognitive functioning and to identify the cognitive domains most responsive to the intervention.

### Barriers to CCT Engagement and Pathways for Functional Adaptation

This study also revealed several negative experiences and practical barriers encountered during CCT. Some children experienced difficulties with particular game-based tasks, especially when task demands were not well matched to their age or cognitive abilities. Consistent with previous research, inappropriate task difficulty may increase frustration and reduce children’s willingness to continue training [37]. This may be partly related to the relatively young age of the participants and the potential mismatch between the developmental characteristics of young children and the task demands of the CCT program. In addition, cultural and linguistic differences may have affected some children’s understanding of game instructions, rules, or scenarios. Hronis et al [38], in their study of children with intellectual disabilities, highlighted the importance of adjusting task complexity according to children’s developmental characteristics. Similarly, previous research has shown that when task difficulty is appropriately matched to individual skill levels, participants may experience greater flow and enjoyment, whereas overly difficult tasks may increase frustration [39]. Future adaptation should therefore extend beyond simple translation to include culturally and developmentally appropriate game narratives, visual materials, instructions, reward systems, and examples. Such adaptations may improve children’s understanding of the activities, reduce unnecessary cognitive burden, and allow them to focus their effort on the intended cognitive training rather than on interpreting unfamiliar content.

Engagement was also shaped by the realities of cancer treatment and family life. Caregivers described occasional absences or interruptions in training, often in the context of fatigue, pain, nausea, treatment appointments, hospitalization, caregiving demands, and competing family responsibilities. These interruptions should not be interpreted simply as a lack of motivation or poor acceptability. Rather, they may reflect the fluctuating physical condition of children undergoing cancer treatment and the substantial practical demands placed on their caregivers. Previous research has likewise shown that computerized cognitive interventions may be feasible and acceptable for populations with pediatric cancer, while participation remains dependent on families’ capacity to integrate training into changing treatment and home routines [21,27]. Flexible delivery models including brief sessions, adjustable schedules, pause-and-resume functions, and reminders that can be tailored to treatment routines may therefore be particularly important for sustaining engagement.

Beyond these routine challenges, families described barriers operating at the social and economic levels. Some parents worried that receiving cognitive training might cause their child to be labeled as “mentally retarded.” This concern appeared to reflect limited understanding of cancer-related cognitive late effects and their distinction from intellectual disability [24]. When cognitive support is associated with developmental or intellectual impairment, families may hesitate to acknowledge cognitive difficulties or seek assistance. This interpretation is consistent with previous research suggesting that awareness of cancer-related cognitive late effects among caregivers remains limited [40]. These findings highlight the importance of improving awareness of cancer-related cognitive late effects at both the family and societal levels. Providing clear information to caregivers about the nature of cognitive late effects and their distinction from intellectual disability may help address misconceptions before CCT is introduced. Broader public education about the cognitive consequences of childhood cancer and its treatment may also help reduce stigma and promote greater understanding of the need for cognitive support.

Economic accessibility represented another important barrier. Some parents described the potential cost of CCT as “several hundred yuan per month” and indicated that they might “wait a few more years” before considering its use. Such responses may reflect the substantial financial burden already experienced by families managing childhood cancer and its treatment [41]. Becker et al [42] further highlighted the importance of economic accessibility in symptom management. Importantly, these barriers may reinforce each other. Even when CCT is made more affordable, families may still hesitate to use it if they fear that the need for cognitive training could lead others to question their child’s abilities or associate the intervention with developmental or intellectual problems. Such concerns may create social embarrassment and further discourage families from seeking cognitive support. Addressing these barriers may therefore require both improved awareness and accessible delivery models, including clear, nonstigmatizing information about cancer-related cognitive late effects and affordable approaches to CCT delivery.

Taken together, the range of barriers and support needs identified in this study suggests that CCT, as a mobile health tool, should provide more than cognitive training alone. Future CCT could incorporate functions that address the broader needs of children and their caregivers, including personalized training tailored to individual cognitive characteristics, flexible training arrangements, caregiver education, training reminders, and ongoing communication with health care professionals. Accessible educational materials, such as brief videos and brochures, could improve caregivers’ understanding of cognitive late effects and promote nonstigmatizing communication, while low-cost subscription options and other scalable delivery models could help reduce economic barriers. Incorporating these functions into CCT may provide more comprehensive and continuous support for children and their families, thereby improving its accessibility, acceptability, and sustainability in pediatric cancer care. Although concerns related to prolonged screen use, such as visual discomfort and excessive reliance on digital devices, were not identified in the present study, future CCT development should nevertheless consider these potential issues and could incorporate appropriate screen-time guidance, scheduled breaks, and reminders for eye rest to promote safer use of CCT.

### Limitations

This study has several limitations that should be considered when interpreting its findings. First, the sample was drawn from a single hospital, which may limit the transferability of the results to other clinical or cultural settings. Nevertheless, this focused recruitment enabled in-depth engagement with parents who had direct experience with CCT, generating rich, contextually grounded insights. Future studies involving multiple sites and more diverse populations would strengthen the applicability of the findings across different care environments. Second, the first author was involved in delivering the intervention and conducting weekly telephone guidance, which may have influenced participants’ responses or encouraged more favorable reports. To address this potential influence, the interviewer encouraged open and candid responses during the interviews. In addition, the first and second authors independently coded the transcripts and subsequently discussed differences in coding and interpretation. Nevertheless, the possibility of interviewer and researcher influence cannot be completely excluded. Third, because this study focused on parents’ perspectives rather than children’s direct experiences, some nuances of pediatric users’ interaction with CCT may not be fully captured. Future research incorporating children’s voices, alongside more varied samples, will offer a more comprehensive understanding of CCT use in pediatric oncology. Fourth, the use of purposive and convenience sampling may have introduced selection bias. Parents who were more willing or available to participate in the interviews may have had experiences or perspectives that differed from those who declined or were unavailable to participate. Therefore, the findings may not fully represent the perspectives of all parents of children with cancer who use CCT. Future studies should consider broader recruitment strategies to capture a wider range of parental experiences and perspectives.

Despite these limitations, this study offers important insights into how families perceive CCT and identifies practical considerations for improving its accessibility, usability, and integration into cognitive rehabilitation practices for children with cancer. These findings lay the groundwork for future research and contribute to the ongoing refinement of child-centered cognitive interventions in pediatric oncology.

### Conclusions

This study provides insight into parents’ perspectives on the use of CCT for managing cognitive late effects in children with cancer. Parents described CCT as practical, engaging, and convenient for home-based cognitive training, while also reporting challenges related to task difficulty, missed training sessions, stigma, cost, and limited information. Some parents perceived changes in their children’s cognitive functioning following CCT; however, these perceptions do not establish intervention effectiveness. Parents also identified a need for individualized training, reliable information, and ongoing professional support. These findings provide practical considerations for refining CCT and exploring its integration into broader mHealth frameworks, including attention to the safe and sustainable use of digital devices. Future research should include more diverse samples, incorporate children’s perspectives, consider potential concerns related to prolonged screen use, and use objective cognitive outcomes to evaluate the effectiveness of CCT.

## Abbreviations

CCT: computerized cognitive training
RATS: Relevance, Appropriateness, Transparency, and Soundness
RCT: randomized controlled trial

## Data Availability

The data that support the findings of this study are available from the corresponding author upon reasonable request.
